# Developing Family-Community Interactive program for the disabled older adults at home: a study protocol

**DOI:** 10.1186/s12877-023-04273-7

**Published:** 2023-09-29

**Authors:** Peng Wang, Meilan Niu, Ying Chen, Shanshan Wang, Chuqiao Wu, Di Zhao, Panpan Wang, Huashan Yang, Panpan Guo

**Affiliations:** 1 Medical School of Huanghe Science and Technology University, Zhengzhou, China; 2 School of Nursing and Health, Zhengzhou University, Zhengzhou, China; 3 Huanghuai University School of Medicine, Zhumadian, China; 4 School of Nursing, The Hong Kong Polytechnic University, Hung Hom, Hong Kong; 5 Henan Cancer Hospital, Affiliated Cancer Hospital of Zhengzhou University, Zhengzhou, China

**Keywords:** Interactive program, Family, Community, Disabled older adults

## Abstract

**Background:**

With an aging population and the influence of traditional culture, the number of disabled older adults at home is increasing. Meanwhile, their care needs are also increasing. The cooperation between family and community can effectively improve the quality of home care for the disabled older adults. At present, there is a lack of research on the interaction between family and community in home care for disabled older adults.

**Methods:**

The aim of this study is to determine the experience and demands of the interaction for disabled older adults, family and community, construct an interaction program among disabled older adults, family and community, and improve the quality of life. From may 2022 to July 2022, This study will select disabled older adults families from seven communities in Henan provinces. The researchers, after training, will conduct semi-structured interview to collect research data. According to the integration results of qualitative research, the interactive program is constructed and revised using the Delphi expert consultation method. Then the participants will be selected to accept the intervention of the interactive program and evaluated through questionnaires.

**Discussion:**

Both family and community play an important role in the care of the disabled older adults at home. There is some evidence indicating the benefits of cooperation between family and community on disabled older adults. This study will take a step further and constructs a interaction program about how to create a positive and interactive home-based older adults care environment.

**Trial registration:**

Registered in the Chinese Clinical Trial Registry on April 19, 2021, number ChiCTR2100045584.

## Background

Population aging has become a worldwide phenomenon, and the concern of older adults care is expanding worldwide [1]. The scale of older people, has been increasing year by year. The seventh census of population in China shows that there are 190 million people aged 65 or above, accounting for 13.5% of the total population [2]. Due to the decrease of mortality rate and the extension of life expectancy, the number of the older adults is increasing at the rate of 5.96 million per year. It is predicted that by 2040, China’s elderly population will reach 400 million [3]. With advancing age, it means the continuous deterioration of various physiological functions. This population trend also means that the proportion of people with mental or physical disabilities who are unable to carry out activities of daily living increases [4]. According to statistics, by 2019, there will be more than 40 million disabled and semi disabled older people in China, which is expected to reach more than 60 million by 2030 and 96 million by 2050 [5].

“Disability” is an integrative concept that represents the state of incomplete self-care due to various reasons such as old age, disease or physical and mental disorders [6, 7]. With the intensification of the aging of the population and the increasing number of disabled older adults, the problem of care for the disabled older people has become increasingly prominent. In China, institutionalization is a choice of care. However, due to the high price and the influence of traditional culture, Family is still the choice of most elderly people. Besides, due to physical weakness, most elderly people with disabilities are more vulnerable to the pressure brought of the new environment [8, 9], and are unwilling to choose to live in unfamiliar environment outside the family and community [10, 11].

To meet the needs of the disabled older adults, the government advocates and encourages home-based care [12]. Both family and community play an important role in the care of the disabled older adults at home. Their mutual cooperation can effectively improve the quality of home care for the disabled older adults [13]. In the context of home-based care, engagement of community can not only meet the demands of the disabled older adults who are eager for stay-at-home, but also help families provide life care and ensure the quality of care for the disabled older adults [14]. Compared with institutional elder care, home elder care has the advantages of low cost and high efficiency, which make the demand for community care services of the disabled older adults continue to increase [15].

At present, the family members, with children and spouses as the main body, are still the main providers of home care for the disabled older adults [16]. In China, women play the role of the main family caregivers. The care tasks of the disabled elderly are generally undertaken by women in the family, such as female spouses, daughters and daughter-in-law [17]. This is similar to other countries in the world [18, 19]. Meinowb et al. [20] found that with the increase of age and disability, the care needs of the older adults at home also increase. However the trend of family’s centralization and miniaturization leads to the decrease of available care manpower in the family. In addition, the burden of long-term care and the lack of time and energy further weaken the function of current care manpower; furthermore, family caregivers are generally deficient in professional caring knowledge and tools, which can not satisfy the requirements of technical care services, resulting in the deficiency of care workforce and ability, which directly affects the quality of care for the older adults [21, 22]. Therefore, in the process of home care for the disabled older adults, the support provided by the family is very limited, and it is difficult to fully realize the long-term care for the disabled older adults. The family’s demand for the assistance of long-term professional care from the community is also growing [23].

Muramatsu et al. [24] found that home-based and community-based care can effectively improve the mental health level of the disabled older adults. Other studies have shown that the establishment of professional community medical and nursing service institutions can take over the care of the disabled older adults when their families can not take care of them, so as to avoid their worries [25, 26]. On the other hand, professional community service institutions can ensure the disabled older adults to seek medical treatment at the first time in case of physical discomfort or emergency, and reduce the treatment risk of the disabled older adults [27]. Community medical staff provide professional and refined services. Family caregivers can replace professionals, provide less technical informal care services. Family and community coordinate and cooperate, which enable the disabled older adults to live in their familiar environment and receive professional care services [28]. The effective interaction between family and community is very important in the process of caring for the disabled older adults at home.

With the continuous promotion of national basic public health services, the number of community nurses in China is increasing. At the end of 2020, there were 21,9574 community nurses in China [29]. Although health and social support such as home sickbed, door-to-door services and respite care based on community institutions can be used to help the disabled and their families, they have not been fully promoted and developed. Many families have low awareness of these health services and are worried that they cannot be reimbursed due to high prices [30]. Meanwhile, Community nurses focus on providing services to the elderly and lack awareness of attention and communication with family caregivers [31, 32]. Community health professionals have not established good collaboration and trust relations with families, and are worried that conflicts may arise and personal safety cannot be guaranteed [33]. Thus, how to form a positive interaction is more of an open question.

**Fig. 1:**
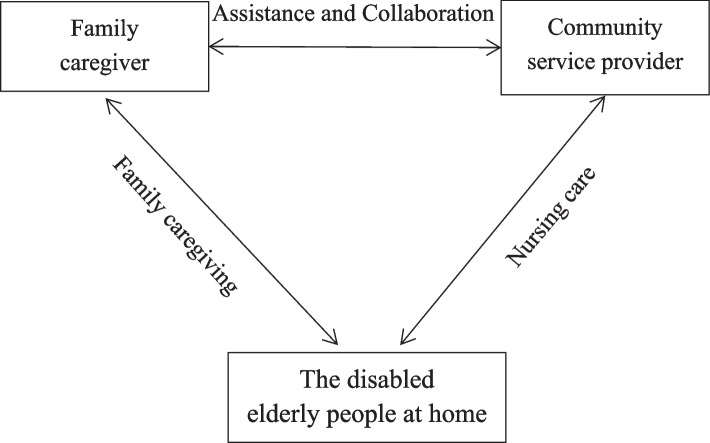
Interaction mechanism among the disabled elderly people, family and community

Given that social relationships with community service providers may serve as coping resources for families, pro-active strategies to facilitate positive collaborative relationships to optimize family–community interactions may be beneficial. A recent review [34] of Ris-Schnepp, and Mahrer-Imhof identified five important factors for community-based family caregivers: “relationship building with professionals” “negotiating with professional care” “being professionally supported” “managing role expectation and knowledge sharing” and “collaborative practice” with home care nurses. And according to Conceptual model by Ris-Schnepp, and Mahrer-Imhof, the interaction mechanism is adapted (See Fig. 1). Family caregivers and community service providers cooperate and interact with each other to provide home care and professional care for the disabled elderly, jointly taking care of the disabled elderly at home.

The effective interaction between family caregivers and community nurse is very important in the process of caring for the disabled elderly at home. However, the previous studies on the interaction relationship between family caregivers and medical staff mostly focused on aging service agencies and hospitals, and did not pay much attention to the contexts of family caregiving in community settings [35–37]. The comprehensive research on the family and community of the disabled older adults at home and the research on the interaction between them are relatively lacking, which is not conducive to improving the quality of care for the disabled older adults at home.

Therefore, the aim of the study is to construct the interaction program between family and community for the disabled older adults at home, based on the theory of social interaction and qualitative research on the families, communities and the older adults with disabilities at home. It is of great significance to strengthen the core function of family, improve the level of community participation, and cultivate the sense of cooperation between family and community.

## Methods/design

**Fig. 2:**
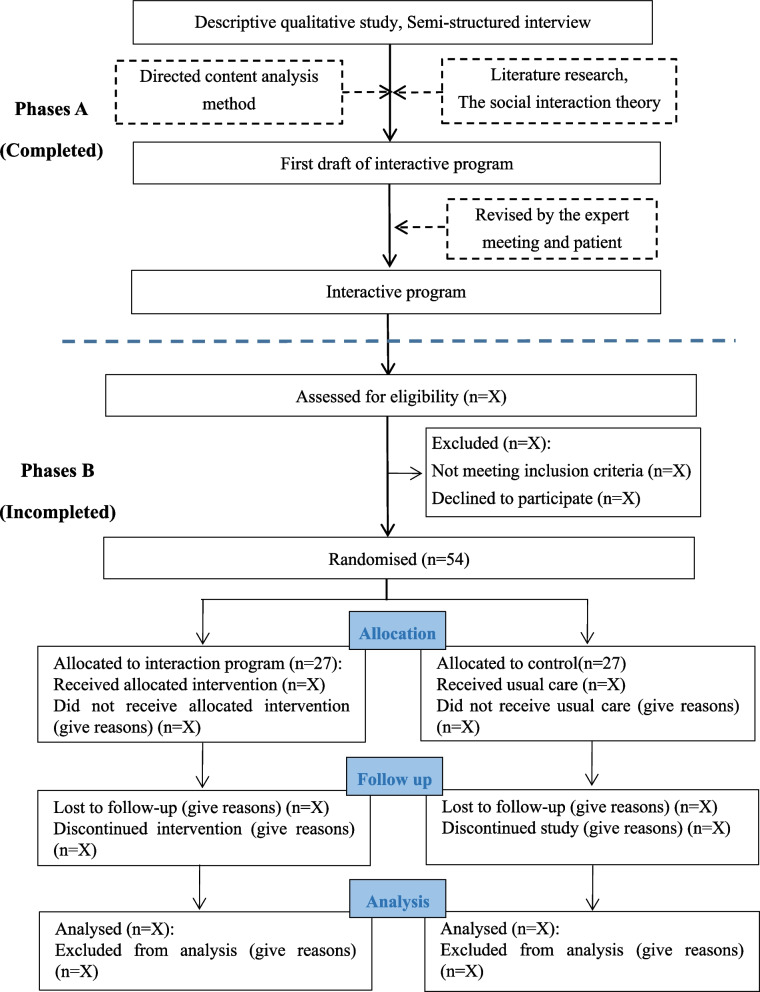
Study flow chart

The study consists of two phases (A and B) (See Fig. 2). The overall goal of this programme of research is to develop and evaluate interaction program between family of disabled older people and community to provide collaborative intervention and management program for disabled older adults at home to increase their quality of life. Specific objectives for each phase of development/evaluation include: (1) develop the program content to construct the interaction program among disabled older people, family and community (phase A), (2) assess feasibility in terms of implementation (accrual rates, acceptability and level of engagement) and determine an initial estimation of effectiveness outcomes in a pilot randomised controlled trial (RCT) (phase B).

### Phases A

The aim was to construct the interaction program between family of disabled older people and community to address the research gap based on the theory of social interaction. Semi-structured interview in Phases A was assisted to: (1) learn about the interaction experiences and care needs of the family caregiver of disabled older people and the community nurse, (2) design content, construct interaction program and use the expert consultation to revise. This program will help realize the positive interaction between family caregivers and community nurses and increase their quality of life of disabled older people.

### Participants

We recruited participants through purposive samplings from the Community Health Services Center in Henan. Firstly we contacted the community nurses and interviewed them after consent. After the interview, community nurses led us to find family caregivers of disabled elderly people who meet the inclusion criteria within their jurisdiction. Explain the purpose and significance of the study, and ask if they are willing to participate in the interview. The study had been approved by the ethical review committee of Zhengzhou University (ZZUIRB2021-15).

### Eligibility criteria

#### Inclusion criteria

The older adults were [38]: aged ≥ 60 years; Katz ADL score < 6; at home for more than 3 months; good communication skills. The inclusion criteria of family caregivers were [39]: aged ≥ 18 years old; the family members of the disabled older adults and taking care of the older adults as the main caregiver for more than 3 months. The inclusion criteria of community service employees were: aged ≥ 18 years old; the service and management personnel of relevant institutions in the community who often have direct contact with the older adults and provide door-to-door services; having been engaged in community service for more than one year.

#### Exclusion criteria

All participants decided not to participate in the present study, included vacationers and temporary employees. And family caregivers who have potential cognitive impairments and fail to communicate.

### Study setting

During phase A, the family caregivers of disabled older adults and community staff were invited to participate in the study by completing interview. Disabled older adults families was selected from seven communities in Henan. Chose the time when the disabled older adults are in good mental state and have free time. The family caregivers of disabled older adults and community nurses were investigated and interviewed separately to ensure that they did not interfere with each other. The interview place was chosen according to the interviewee’s wishes, and was usually set in the house of the disabled elderly people or community meeting room.

### Procedures

Semi-structured interview [40] is a research method in which the interviewer contacts the interviewee, carries out purposeful conversation and dialogue according to the interview outline, and then collects data. From may 2022 to July 2022, this study interviewed family caregivers of disabled older adults and community nurses according to the interview outline, explored their experience and needs of interaction between family and community for disabled older adults, and provided reference suggestions for the formulation of interactive treatment plan. For qualitative data, the audio-taped interview was listened repeatedly and transcribed verbatim. Two investigators used directed content analysis method to work on the data analysis [41]. Before the formal analysis, the interviewees were coded with letters A-H to process personal sensitive information such as their names. Then directed content analysis method was used to extract the effective content, code, classify and simplify the relevant content, extract the theme and return to the interviewees for confirmation, and finally refine the interview theme. The qualitative study results have been reported [42]. The interactive items of interaction program were formed based on the interactive experience and needs, literature research [43] and the social interaction theory [44, 45]. Then expert consultation [46] was used to modify interaction program. Experts in psychological nursing and management, demography, sociology and other fields were invited to suggest modifications and provides theoretical and technical guidance. The expert consultation was divided into three rounds, including one round of online one-to-one open expert consultation and two rounds of Delphi expert consultation. According to Likert’s 5-level scoring method, an opinion column were set up for experts to fill in their opinions. In addition, stakeholder groups (including disabled older adults, family caregivers and community workers) also was invited to provide suggestions for the revision of program.

## Results

**Fig. 3:**
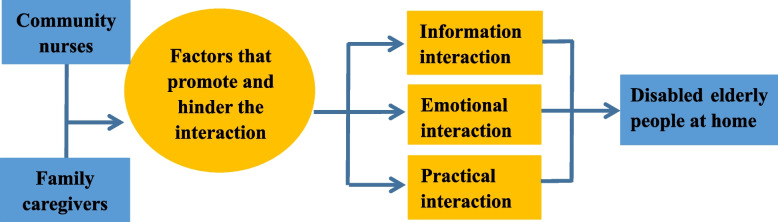
The interaction between community nurses and family caregivers of disabled elderly

A total of 12 interviews were completed, including 7 family caregivers and 5 community nurses. Based on the data collection and directed content analysis, four overarching themes were identified: Information interaction, Emotional interaction, Practical interaction, and Factors that promote and hinder the interaction. And we redefined the interaction mechanism between community nurses and family caregivers for disabled elderly people. Provided guidance for the theme and content of the interactive program (See Fig. 3).

Finally, we collated the findings of the qualitative interview, the expert consultation and stakeholder feedback to finalise the protocol for the feasibility study and model the intervention. The intervention strategies of the interaction program consisted of eight major items (8-item-interaction program, See Table 1).

**Table 1:** Interaction program between family caregivers and community nurses for disabled elderly people

**Item**	**Objective**	**Content**
Skill training	The training for community nurses and family caregivers in communication skills and home care for the disabled elderly people is an important guarantee to promote effective interaction.	**Training participants: community nurses and family caregivers** **Training content:** **① Communication skills training.** The content includes community nurses and family caregivers, sharing information about illness care, creating an atmosphere of trust, and using verbal and non-verbal interaction skills such as positive listening, empathy and physical contact. **② Home care training for the disabled elderly person.** Including disabled elderly limb joint movement, emergency management, prevention and treatment of infection problems, pipeline care, drug use and side effects, feeding or assistance in eating, turning over and skin care, body cleaning, and oral cleaning.
Establish a partnership	Community nurses build trust and good partnerships with family caregivers in order to promote effective interaction.	① The community nurse first arrived at the home of the disabled elderly person and communicated with the caregiver. ② Introduce the theme of this intervention, tell about the main content of the intervention program, and ask the caregivers’ willingness to participate and their views and expectations of the interactive program. ③ Establish an interactive wechat group to facilitate community nurses and caregivers to contact with each other and promote effective interaction.
Evaluation and planning	Assess care issues for disabled elderly people and their family caregivers. Work with family caregivers to develop care plans and clarify interactive content.	① Community nurses firstly assess the health problems of the disabled elderly and the problems and difficulties in the care of the caregivers. ② Caregivers take the initiative to discuss with community nurses how to address the current health issues of disabled elderly people, and jointly develop long-term and short-term care goals and plans for disabled elderly people.
Division of labor and cooperation (Behavioral interaction)	Community nurses and caregivers negotiate the division of labor and cooperation on the care issues and care plan of the disabled elderly, define their respective roles, functions and care priorities, and implement the care plan.	Community nurses and caregivers practice the division of labor and cooperation in the care plan of the disabled elderly, which is a practical behavioral interaction. **The roles of community nurses mainly include:** basic nursing, health education and consultation, cooperation and coordination, supervision and observation, and **the role functions of caregivers mainly include:** care for daily life and activities, some technical care (condition observation, assistance with medication taking, turning and back), mental and emotional support.
Information interaction	Community nurses and family caregivers exchanged information through information sharing communication.	① The caregivers shared the daily and health conditions of the disabled elderly, as well as the problems of the caregivers with the community nurses in the WeChat group. ② Community nurses give timely feedback and response, and ensure that they answer caregivers’ care questions every day. ③ Community nurses push popular science articles or videos on health and care knowledge of disabled elderly once or twice a week, and can discuss and learn with caregivers to ensure that the learning content is easy to understand, simple and intuitive. ④ The community nurses informed the caregivers in time of the physical examination and lectures in the community health service center.
Emotional interaction	Through talking and listening, community nurses and caregivers can further close the distance between each other, resonate, and promote the emotional interaction between community nurses and caregivers.	① **pour out.** Community nurses can timely pay attention to caregivers in the process of long-term care of negative emotions, guide the caregivers to express and vent years backlog of suffering, encourage caregivers to share 1–2 is the most difficult levels, release depressed emotions, allowing caregivers to express by talk, complain, cry, release pressure; ② **listen for.** Community nurses can give appropriate comfort and encouragement, and can use various verbal and non-verbal methods such as listening, empathy, and physical contact to provide psychological and emotional support for the caregivers, and tell the caregivers that "I am there". Actively encourage caregivers to develop personal hobbies, enrich their daily life, and maintain an optimistic attitude.
Supervision and feedback	During the implementation of the care plan, supervision and feedback can ensure the smooth progress of the care plan.	Through telephone or family follow-up, community nurses can receive timely feedback from caregivers on family care, timely find out the problems and deficiencies in the process of caregivers’ care, give corrections, and provide caregivers with guidance on care skills.
Evaluation and re-evaluation	The effect was evaluated according to the goal of care, re-evaluated, and the interactive program was continued.	Family caregivers and community nurses together evaluate the implementation of the care plan through telephone or family visits, reevaluate the solution degree and needs of the health problems of the disabled elderly and their caregivers, summarize the problems in the implementation of the plan, discuss the solutions or make the modification and adjustment of the plan.

### Phases B

In B phase, this is a two-group parallel single-blind pilot RCT. Interaction program will be introduced on family caregivers of disabled older adults and community nurses to evaluate its operability and effect. Control group will be given routine home care and intervention group will accept the care of interaction program.

### Participants

Same as Phases A.

### Eligibility criteria

Inclusion/exclusion criteria have been previously described (phases A). Additional exclusion criteria will include participants who participated in phase A study.

### Study setting

Participants will attend one in-person session to learn about the trial, obtain informed written consent and complete demographic and baseline measures (T1). Participants allocated to the intervention group will accept interactive program intervention. The intervention place is chosen according to the interviewee’s wishes, and is usually set in the house of the disabled elderly people or community.

### Procedures

Following ethics approval, the study will recruit participates using the methods described previously (phases A). Eligibility criteria will be confirmed, verbal consent obtained and an appointment for an initial study visit will be made. We will use a variety of methods to promote recruitment and retention, such as giving small gifts during the study and regular follow-up monitoring. The interaction program will be burdensome for participants of intervention group, which we will assess in our process evaluation.

In this study, two communities with similar scale, environment, culture and configuration were selected conveniently, and family caregivers of disabled elderly people and community nurses who met the inclusion criteria will be admitted by the cluster. Following completion of baseline measures, participants will be randomised to the control or intervention group by coin toss. Participants allocated to the control group will receive the usual care and supports, including usual caring appointments and follow-up. Then, participants randomised to the intervention group will consist of use of the interaction program intervention in addition to usual care. It is not possible to blind the participants to group allocation due to the specific nature of the intervention; however, the data analyst who is blinded to treatment allocation will conduct the analysis ensuring neutrality of the outcome assessment.

### Outcomes

#### Qualitative interview

In Phase B, up to 20 in-depth interviews will be conducted with about 10 study participants(agreed to participate) and 10 participation refusers (refused to participate) purposefully selected from the quantitative study sample to represent a variety of gender and ages to explore experiences with admission or rejection of study participation.

#### Questionnaire measurement

In B phase, outcomes of collaboration, quality of life, family and community support will be measured using questionnaires on disabled elderly people, their family caregivers to evaluate the interaction program.

1. General information questionnaire

The general information questionnaire is designed in combination with literature research, which mainly is used to collect the social demographic data of the all participants.

(2) Family Participation in Professional Care scale [47]

A ten-item Family Participation in Professional Care scale was used to assess the extent to the collaboration between family members and community service providers. A 5-point Likert scale was used to assess collaboration in planning, providing care, information exchange, identifying resources, making decisions, and evaluating the effectiveness of care strategies (α = 0.92 for family caregivers; α = 0.79 for service providers). Responses were never (1), rarely (2), sometimes (3), often (4), and consistently(5). An average score was calculated for each participant to indicate overall levels of collaboration.

(3) Katz Index of Independence in Activities of Daily Living (ADL):

This tool was developed by Katz et al. in 1959, which includes of 6 Basic ADL skills including: bathing, dressing, transfer, toileting, feeding and continence. The score for each activity is varied between(unable to do activity) and 1(able to do activity) so that the total score of this tool varies from 0 (lack of performance) to 6 (maximum performance). A score 6 represents the full function, 4 represents moderate impairment, and 2 or less represents severe functional impairment [48, 49].

(4) The Medical Outcomes Study 36-item Short Form Health Survey (SF-36):

The SF-36 survey is a widely used assessment instrument which consists of 8 dimensions: physical functioning (PF), role limitations relating to physical health (RP), bodily pain (BP), general health perception (GH), vitality (VT), social functioning (SF), role limitation relating to emotional health (RE) and mental health (MH). It includes two comprehensive assessments, i.e., physical health (including PF, RP, BP, and GH), and psychological health (including VT, SF, RE, and MH). The score of each domain ranges from 0 to 100, with higher scores indicating a better-perceived quality of life. The Cronbach’s α coefficient of the scale is 0.869 [50, 51].

(5) Family Support Questionnaire (FSQ)

The 15-item FSQ (Chinese version) will be used to measure the level of family support, which was developed by HaiYan Zhang revising Percaived Social Support Family Scale (PSS-Fa) in 1999. A 0–1 rating scale is used for each item, with higher scores representing more family support. The scale showed good reliability and validity when used in the Chinese population, and Cronbach’s alpha was 0.95 [52–54].

(6) Social Support Rate Scale (SSRS)

Shuiyuan Xiao developed this tool in 1986. The tool consists of ten items in three dimensions including objective support, subjective support and utilization of social support. The higher the total score of it, the higher the social support [55]. Cronbach’s alpha of the scale was 0.81 and the test-retest reliability was 0.92 [56].

### Sample size

Using the quality of life of disabled elderly people as the primary outcome, the sample size was calculated by comparing two means. We adopt the sample size estimation formula: N1 = N2 = 2[б (t α + t β)/ (u1-u2)]^2^, α = 0.05, β = 0.10, *t *_*α*_ = 1.96 and *t *_*β*_ = 1.28. Then, according to the literature [57], we know u1-u2 = 13.77 and б = 13.89. Therefore, the sample size of each group is about 22. However, considering the loss of follow-up rate of 20%, we will need a total sample size of 54 (27*2).

### Data management

Data will be collected through questionnaires by the researchers in the survey, which will be strictly preserved by their assistants.

### Statistical methods

The quantitative data will be entered using Epi-Data 3.1 by two person and analyzed using SPSS version 20.0. Firstly, data is summarized using descriptive statistics. Then the mean ± standard deviation (x ± s) is used to describe the measurement data, and the rate and constituent ratio of count data are used to describe the statistics.

### Validity and reliability

This research programme is led by a senior researcher with rich research experience in the field of the older adults. As for the qualitative data, the transcribed verbatim was verified against the taped interview by two people. Content analysis will be conducted with qualitative data software. The quantitative data was entered by two person for validation purposes and analyzed by professional statistician to reach high level of the quantitative results. All data collection tools and outcome measures have demonstrated good reliability and validity.The credibility of the data analysis will be kept by carrying out regular reporting to the research team.

## Discussion

With the deepening of aging population degree, long-term care for disabled older adults has become a challenging problem gradually. Home and community-based care service can combine the advantages of both good family care and community professional services [28]. Some studies have shown that creating a positive and friendly social environment based on the theory of social interaction can promote the positive interaction between family and community, effectively improve the quality of care for the disabled older adults at home, and reduce the burden of family and society [58–60].

Studies have reported the importance of the quality of social interactions between family and formal care providers in residential care settings, and less is known about such relationships in community-based care settings in which the majority of disabled older adults [61, 62]. It is known that there are many problems in the interaction between family and community in the process of home care for the disabled older adults [22, 37]. To our knowledge, similar studies are relatively sparse in the international evidence. To solve these problems, this study takes a step further and to elucidate the underlying program about interaction between family caregivers and community employees.

The social interaction theory [44, 45] was added as the theoretical basis of this project. This study constructed the interaction program of family-community for the disabled older adults at home to promote the formation of benign interaction between family and community, which not only will meet the care needs of the disabled older adults at home, improve their quality of life, but also enriches the social interaction theory and solve some deficiencies in the current research (that is, the lack of benign interaction). It is of great significance that the project will, for the first time, describes the interaction program between family and community in the context of the disabled older adults.

It is very valuable to study the interaction between family and community of the disabled older adults at home. Collecting qualitative data can help us analyze their interaction bettween family-community from a special perspectives and enable us to accurately construct their interaction program. The formulation of an interaction program can strengthen the collaboration among disabled older adults, families and communities, and the timely feedback from family members and patients can help community staff provide more effective care [54]. This in turn will contribute to the development and dissemination of interaction among patients, family and community.

The study developed interaction intervention that can be used for the disabled older adults in community settings. Two disabled older adults, their family caregivers and community staffs as representatives will be members of research team to comment on study design including topic guides for the qualitative interviews to ensure that all aspects of the study are disabled older adults focused. And they will continue to be actively involved in development and revision of interaction program. Finally, the results will be disseminated to the participants in the form of a lecture and fellowship at the end of the study. If the program proves to be viable, it will be offered and guaranteed to the participants.

As described, we see the potential of the constructing of interaction program, which can solve the problem of home care for the disabled older adults, and be in line with the wishes and needs of the disabled older adults. The interaction between family caregivers and community nurses will be very beneficial, which will reduce the burden of family caregivers and have positive impacts on the disabled elderly people through better care provision.

### Strengths and limitations

Qualitative study was used to explore the interaction experiences between family caregivers and community nurses for disabled elderly people at home. We collected the voices and feelings about interaction directly from participants to develop the interaction program. Despite this study’s important contribution to home care of disabled elderly people, two limitations should be mentioned. The conduct of our study has been greatly affected due to the COVID-19. There were some difficulties in recruiting and contacting eligible participants. Therefore, the study had small samples, which limits the generalizability and representativeness. The sampling method may have resulted in selection and response bias. Family caregivers were recruited via purposive sampling through community nurses. Those participants were more likely to have a better relationship with community nurses. We communicated with community nurses in advance, in order to avoid having their present which could cause response bias. However, in a few cases, community nurses were present for a portion of the interview, and family caregivers could have felt more inclined to give a positive evaluation of interaction with community nurses.

## Data Availability

Not applicable.

## Abbreviations

ADL: Activities of Daily Living
SF-36: The Medical Outcomes Study 36-item Short Form Health Survey
FSQ: Family Support Questionnaire
PSS-Fa: Percaived Social Support Family Scale
SSRS: Social Support Rate Scale
